# Developmental regulatory genes in recalcitrant forest trees: advances in somatic regeneration and genetic transformation

**DOI:** 10.3389/fpls.2025.1689705

**Published:** 2025-11-14

**Authors:** Ying Wang, Jing-han Wang, Pu-rui Guo, Jing Peng, Chun-ze Xie, Yi-dan Shi, Yuan-hang Wu, De-zhi Liao, Song Sheng

**Affiliations:** 1 College of Forestry, Central South University of Forestry and Technology, Changsha, China; 2 State Key Laboratory of Utilization of Woody Oil Resource, Central South University of Forestry and Technology, Changsha, China; 3 Institute of Forestry, Hunan Academy of Forestry, Changsha, Hunan, China; 4 Yuelushan Laboratory, Changsha, China

**Keywords:** recalcitrant forest species, somatic regeneration, genetic transformation, developmental regulatory genes, genomics, plant biotechnology

## Abstract

Forests play a pivotal role in maintaining global ecological balance, supporting economic development, and mitigating climate change. However, many economically and ecologically important tree species—particularly long-lived, highly heterozygous, and genomically complex taxa—remain notoriously recalcitrant to efficient clonal propagation and genetic transformation. Major constraints include low somatic regeneration capacity, strong genotype dependence, and limited regeneration of transgenic tissues, all of which impede rapid breeding and practical deployment. In recent years, developmental regulatory genes (DEV genes), which govern cell fate reprogramming and facilitate regeneration, have emerged as key molecular targets for overcoming these technical bottlenecks. This review provides a comprehensive synthesis of recent advances in the identification and functional characterization of DEV genes in model systems and crop species, with an emphasis on their translational potential in recalcitrant forest trees. We highlight strategies for leveraging DEV-mediated regulatory mechanisms to enhance somatic regeneration and transformation efficiency, and propose tailored application frameworks for forestry species. Ultimately, the integration of DEV gene-based approaches may offer a robust theoretical and technological foundation for the accelerated breeding, large-scale propagation, and germplasm conservation of elite forest genotypes, thereby contributing to the long-term sustainability of forest ecosystems.

## 1 Introduction

Global forest resources are fundamental to maintaining ecological balance and combating climate change. Beyond their critical environmental roles, forests supply timber, cellulose, and other economically valuable materials essential for human development. In the face of increasing demand for wood products and the escalating urgency of climate-related challenges, accelerating the genetic improvement of forest trees has become a pressing priority. The goal is to develop fast-growing, high-yielding, and stress-resilient elite varieties suited to diverse ecological conditions. However, conventional breeding strategies—such as hybridization and traditional vegetative propagation—are inherently constrained by the biological characteristics of trees, including long generation times, late sexual maturity, highly heterozygous and complex genomes. These limitations result in low breeding efficiency and extended cycles, hindering the timely development and deployment of superior genotypes to meet the dual demands of sustainable production and environmental conservation.

Against this backdrop, modern biotechnological advances—centered on plant tissue culture and genetic engineering—offer unprecedented opportunities for rapid propagation and targeted genetic improvement in forest trees. These technologies enable the clonal multiplication of elite genotypes and the precise manipulation of desirable traits through gene introduction or modification, thereby significantly shortening breeding cycles and enhancing forest productivity and stress resilience. For instance, the development of a high-efficiency root transformation system in woody plants has facilitated the visualization and functional analysis of organelle activities, paving the way for stable tissue-specific transgenesis and long-term subcellular tracking (Gong et al., 2024). Similarly, protoplast-mediated genome editing and regeneration systems have proven effective in recalcitrant tree species such as Larix, enabling precise gene editing without foreign DNA integration and demonstrating the feasibility of non-transgenic improvement strategies (Ma et al., 2024). Moreover, recent progress in combating tissue browning during *in vitro* culture of economic woody plants has addressed a long-standing technical bottleneck in forestry regeneration pipelines, thus improving the viability and scalability of tissue culture systems for elite genotype propagation (Liu et al., 2024).

Extensive research across woody plant species further suggests that *in vitro* propagation techniques can effectively overcome the inherent limitations of traditional vegetative propagation (Shinde et al., 2022), expanding the scope for precise trait enhancement—especially when integrated with gene transfer and regeneration protocols tailored to various developmental stages (Isah, 2023). Although the long-term phenotypic stability of transgenic trees with enhanced resilience and growth traits under field conditions remains to be fully validated, the advent of gene-editing tools such as CRISPR-Cas9 has greatly expanded the prospects for precise genetic improvement in forestry species (Ahuja, 2021; Giri et al., 2004). Additionally, optimized tissue culture systems for hybrid *Quercus alba* have helped resolve trait segregation issues associated with seed propagation, enhancing the uniformity and performance of plantation materials (Sax et al., 2019). Micropropagation protocols established for species such as *Acacia* have further demonstrated the scalability and applicability of tissue culture to large-scale forestry improvement programs (Vengadesan et al., 2002). Collectively, these studies underscore the indispensable role of plant tissue culture and transformation technologies in meeting the growing demand for improved tree planting stock and call for further refinement and integration of emerging biotechnologies to fully realize their potential in sustainable forestry.

## 2 Biotechnological constraints in somatic regeneration and genetic transformation of forest trees

Plant somatic regeneration, as a manifestation of cellular *totipotency*, refers to the ability of differentiated somatic cells to dedifferentiate, proliferate, and subsequently redifferentiate into a complete plant under appropriate *in vitro* conditions. This process typically occurs through organogenesis (via the formation of adventitious shoots or roots) or somatic embryogenesis. Somatic regeneration is a critical step in genetic transformation, as the successful development of transgenic plants requires that cells incorporating foreign DNA regenerate into fertile individuals through efficient developmental pathways. The molecular basis of totipotency and cell fate reprogramming has emerged as a central focus in plant biology, owing to its profound implications in plant breeding and biotechnology (Zhang et al., 2020). Studies in *Arabidopsis thaliana* have revealed that histone-modifying enzymes—such as Polycomb group proteins and Jumonji C-domain demethylases—play pivotal roles in establishing totipotency and enhancing cellular plasticity, highlighting the complexity of epigenetic regulation during developmental reprogramming (He et al., 2021). Transcriptomic analyses of protoplast cultures in *Arabidopsis* further demonstrate that chromatin remodeling and histone variant expression serve as early molecular indicators of dedifferentiation and reentry into the cell cycle, emphasizing the dynamic interplay between transcriptional regulation and the cellular environment in reactivating totipotent potential (Chupeau et al., 2013).

However, many important forest tree species—particularly long-lived, woody plants with complex, highly heterozygous genomes—face persistent and well-documented obstacles to somatic regeneration and genetic transformation, collectively referred to as *recalcitrance*. These challenges are most prominently manifested in the form of low regeneration efficiency and strong genotype dependence, whereby distinct genotypes exhibit dramatically different regenerative capacities under identical culture conditions, and the majority of genotypes fail to regenerate altogether. For example, studies in *Gossypium hirsutum* have revealed substantial transcriptomic differences between highly embryogenic and recalcitrant genotypes, underscoring the importance of transcription factor activity and alternative splicing events in determining embryogenic competence (Guo et al., 2020). Multiple factors contribute to this recalcitrance, including species-specific traits, genotypic variation, the composition of plant growth regulators, and the physical and chemical properties of the culture environment. The interplay of these variables reflects the inherent complexity and multifaceted nature of plant regeneration systems and highlights the intricate cellular and molecular mechanisms underlying totipotency (Chen et al., 2024; Long et al., 2022). As Fehér emphasized, the conceptual and mechanistic understanding of dedifferentiation, callus formation, and totipotency remains incomplete, further complicating efforts to design universally applicable regeneration protocols (Fehér, 2019). These constraints are particularly acute in forest trees, where the combination of biological complexity and limited regenerative plasticity poses a major bottleneck to the application of plant biotechnology (**Table 1**).

**Table 1:** Restrictions and preliminary application plans of biotechnology for different trees.

Species/group	Primary regeneration bottlenecks	Potential molecular/physiological causes	Current solutions/attempts involving DEV genes
Pinaceae *(Conifers)*	Callus induction is difficult; low somatic embryogenesis (SE) frequency; high incidence of abnormal embryos; low maturation rate	Imbalance in endogenous hormones (e.g., elevated ABA); epigenetic regulation (DNA methylation, histone modifications); ROS dysregulation	Application of small molecules (e.g., antioxidants, epigenetic inhibitors) significantly enhances SE efficiency; large-scale application of DEV genes *(WUS/WOX/KNOX)* has not yet been implemented (Guo et al., 2024).
Eucalyptus *(Fagaceae)*	Unstable regeneration systems; low subculture efficiency; regeneration of mature embryos is difficult	High accumulation of phenolic compounds; poor hormone responsiveness; strong genotype dependence	SE systems have been established with initial efforts toward gene transformation platforms; SE induction from mature leaves attempted, but transformation efficiency remains low (Andrade et al., 2011)
Populus *(Poplar)*	Significant SE efficiency differences among genotypes; severe callus browning; low transformation efficiency	High lignification; imbalance in endogenous auxin/cytokinin ratios	Regeneration-associated regulatory genes and allelic diversity identified; allele-specific transcription of regulatory genes proposed as key breakthrough (Ding et al., 2023)
*Larix kaempferi*	Difficulty coupling gene editing with regeneration systems; low replantation rate of edited plants	Weak redifferentiation response; imbalance between endogenous hormones and ROS	DNA-free RNP bombardment combined with optimized SE maturation; superimposed cell cycle/DEV regulation (e.g., CDKB module) (Amiard et al., 2024)
*Malus domestica*	Low explant regeneration rate and narrow transformation window	Hormone signaling and insufficient activation of meristem-related genes	Overexpression of AIL/PLT-like DEVs (such as *MdAIL5*) can reshape hormone and meristem programs and increase bud differentiation rates; further optimization is required by combining the SPL/miR156 pathway (Xing et al., 2023).
Multiple species (review)	Long life cycles; regeneration strongly influenced by genotype and tissue origin; low frequency of transgenic plant recovery	Epigenetic barriers; cell fate reprogramming governed by complex transcriptional networks	Strategies proposed include small molecule application, CRISPR-based editing, and multi-gene regulation (Giri et al., 2004; Nagle et al., 2018)

At the molecular and physiological levels, disruptions in endogenous hormone homeostasis, epigenetic modifications (such as DNA methylation and histone modifications), and insufficient or dysregulated expression of specific developmental regulatory genes (DEV genes) are increasingly recognized as central constraints underlying these technical bottlenecks. Emerging studies have begun to reveal the promise of small-molecule regulators—such as epigenetic inhibitors and antioxidants—in enhancing somatic embryogenesis efficiency. However, the integration of these findings into scalable, reproducible strategies for forest tree improvement remains an unresolved and urgent challenge.

## 3 Harnessing DEV genes to overcome bottlenecks in the breeding of recalcitrant forest species

### 3.1 Functional roles and cross-species potential of DEV genes

Reprogramming somatic cells toward a regenerative state through targeted activation of transcriptional networks that control cellular totipotency has emerged as a promising strategy to overcome regeneration bottlenecks in plants. Among these approaches, the ectopic expression of DEV genes has shown considerable potential. In *Medicago truncatula*, overexpression of *MtWOX9-1*—a *WUSCHEL*-related homeobox gene—markedly enhanced somatic embryogenesis by upregulating downstream signaling components such as *MtCLE08*, *MtCLE16*, and *MtCLE18*, thereby establishing a molecular foundation for efficient plant regeneration (Efremova et al., 2022). In *Nicotiana tabacum*, co-expression of *Arabidopsis BABY BOOM* (*BBM*) and *WUSCHEL* (*WUS*) induced hormone-independent cell differentiation, eliminating the need for exogenous plant growth regulators and improving regeneration in otherwise recalcitrant genotypes (Sato et al., 2024). A similar mechanism was demonstrated in *Jasminum sambac*, where overexpression of *JsWOX1* and *JsWOX4* significantly promoted callus proliferation and root regeneration, highlighting the evolutionary conservation of *WOX* family transcription factors in regulating *de novo* organogenesis across divergent lineages.

Beyond enhancing regeneration, DEV genes have also been successfully applied to improve transformation efficiency in economically important crops. In *Manihot esculenta* (cassava), overexpression of *Arabidopsis GRF5* and the *GRF4–GIF1* chimeric complex accelerated shoot regeneration from explants and substantially increased transformation efficiency, even in highly recalcitrant genotypes (Sato et al., 2024). This *GRF–GIF* overexpression strategy has since been extended to *Beta vulgaris*, *Brassica napus*, *Glycine max*, and *Helianthus annuus*, demonstrating its broad utility in overcoming regeneration barriers across both monocot and dicot species (Kong et al., 2020). Further evidence from cassava shows that manipulation of *LEC1* and *LEC2* homologs facilitates genotype-independent somatic embryogenesis, underscoring their key role in initiating embryogenic competence (Brand et al., 2019). In *Malus domestica* (apple), overexpression of *MdAIL5* enhanced adventitious shoot regeneration through modulation of endogenous hormone levels and activation of stem development-related genes, illustrating the central role of hormonal crosstalk in DEV-mediated regeneration (Liu et al., 2023). Recent transcriptomic analyses have elucidated complex gene regulatory networks that underlie wound-induced callus formation and organogenesis. Key transcription factors such as *PLT3*, *ESR1*, and *HSFB1* have been identified as central integrators of hormonal and wound-derived signals, coordinating transcriptional reprogramming to facilitate cellular fate transitions (Ikeuchi et al., 2018).

Comparative analyses indicate that research on DEV genes spans from model species such as *Arabidopsis thaliana* to a broad range of angiosperms—and, in some cases, even to non-plant systems. Most studies have focused on transcription factors involved in organogenesis, regeneration, stress adaptation, and trichome development. Notably, master regulators such as the *WOX*, *BBM*, *WUS*, and *GRF–GIF* families are consistently highlighted for their conserved roles in overcoming regeneration constraints across taxa. Particularly well-documented is the evolutionary conservation of developmental pathways related to floral and trichome morphogenesis, which supports the notion that core DEV gene functions are transferable across species boundaries. Nevertheless, despite extensive documentation of evolutionary constraint, functional studies in non-model organisms remain limited. This highlights a significant knowledge gap in applying DEV-based strategies to recalcitrant taxa such as oaks and other members of the *Fagaceae* family (**Table 2**).

**Table 2:** Comparison of the research status and application scenarios of DEV genes among different plants.

Author/year	Plant species	DEV key gene points	Applied research	Experimental results/applications
Yan et al. (2023)	Various (overview)	Multiple DEV genes (*WOX*, *BBM*, *WUS*, *GRF-GIF*)	Genotype-independent transformation, regeneration	Reviewed the use of DEV genes (and nanoparticles) to overcome genotype dependence and improve transformation efficiency in diverse species (Yan et al., 2023).
Xu et al. (2024)	Various (overview)	Key developmental regulators (e.g., *WUS*, *BBM*, *GRF*s)	Enhancing regeneration and transformation	Summarized advances in DEV gene application for genetic transformation and regeneration efficiency (Xu et al., 2024).
Davila-Velderrain et al. (2014)	18 angiosperms	Floral organ specification network	Evolutionary conservation	Showed strong purifying selection in floral developmental GRNs, indicating evolutionary conservation across species (Davila-Velderrain et al., 2014).
Yruela (2015)	Various (overview)	Transcription factors, microRNAs	Broad developmental processes	Highlighted conserved yet flexible regulation of developmental processes across plant species (Yruela, 2015).
Valverde et al. (2018)	Various (overview)	GRNs in multiple developmental pathways	Systems biology perspectives	Emphasized integrated omics approaches to study evolutionary dynamics of developmental regulation (Valverde et al., 2017).
Martín et al. (2016)	Various (overview)	Cell fate-determining genes and downstream GRNs	Organogenesis and morphogenesis	Discussed modularity and core TFs controlling developmental transitions, relevant for regeneration (Martín et al., 2016).
Guo (2024)	Various (overview)	GRNs shaping morphological traits	Morphological evolution	Reviewed evolutionary adaptability of GRNs, useful for DEV gene applications in breeding (Guo, 2024).
Doroshkov et al. (2019)	*Arabidopsis* and others	Trichome GRN	Evolutionary development of epidermal structures	Demonstrated protein structure variation and GRN specialization across plant taxa (Doroshkov et al., 2019).
Singh et al. (2018)	Various (overview)	Differential GRNs	Development and disease	Highlighted differential regulation mechanisms relevant to plant stress responses and regeneration (Singh et al., 2018).
Davidson et al. (2022)	Sea urchin (non-plant)	Evolution of dGRNs	Developmental transitions	Provided comparative insights from animal systems relevant for cross-kingdom understanding (Davidson et al., 2022).

### 3.2 Multi-omics strategies for identifying DEV genes in recalcitrant forest species

Identifying effective DEV genes is essential for overcoming regeneration barriers in recalcitrant forest species. Given the inherent complexity of these woody taxa—characterized by long generation times, strong genotype dependency, and high heterozygosity—DEV gene discovery requires an integrative approach that draws on both multi-omics platforms and insights from model and regeneration-competent species. Comparative transcriptomics has proven particularly powerful in this context. By comparing high- and low-regenerating genotypes, or by profiling differential gene expression across key regenerative phases—such as callus induction, proliferation, shoot organogenesis, and elongation—researchers can identify candidate DEV genes with stage- and genotype-specific expression patterns. For example, in *Quercus suber*, comparative transcriptome analysis during somatic embryogenesis revealed numerous differentially expressed transcription factors, including *AINTEGUMENTA*-like, *PLETHORA*, and *AUXIN RESPONSE FACTORs*, providing valuable leads for downstream functional validation (Capote et al., 2019). The emergence of single-cell transcriptomics has further expanded the resolution at which gene expression and cell fate transitions can be studied. This approach enables precise mapping of cellular heterogeneity and identification of cell-type-specific DEV gene activity at critical regenerative stages (Singh et al., 2024).

In parallel, epigenomic profiling offers a complementary dimension to DEV gene discovery. Integrating transcriptome data with DNA methylation (via whole-genome bisulfite sequencing, WGBS), histone modification (ChIP-seq), and chromatin accessibility (ATAC-seq) analyses can reveal dynamic epigenetic reprogramming events that modulate regenerative capacity. In *Q. suber*, for instance, increased somatic embryogenesis (SE) efficiency was correlated with global DNA demethylation levels, and treatment with the demethylating agent 5-azacytidine significantly enhanced SE output—suggesting that epigenetic regulation plays a pivotal role in activating DEV gene expression and promoting regeneration (Carneros et al., 2024). Genome-wide association studies (GWAS) and quantitative trait locus (QTL) mapping have also been applied to identify DEV gene candidates linked to regenerative traits. By correlating phenotypic variation in SE or regeneration efficiency with genomic variation across diverse populations, key regulatory loci can be pinpointed. In hybrid *Populus*, for example, allelic variation at specific loci was shown to influence SE efficiency, highlighting the utility of population-level genetic approaches for uncovering candidate DEV regulators (Ding et al., 2023). Gene family-based approaches provide an additional layer of insight. Known DEV genes from model species such as *Arabidopsis* and regeneration-competent trees like *Populus* can be used as references to conduct homology searches and classify gene family members in recalcitrant taxa. For instance, *GRAS* family members in *Pinus* have been implicated in somatic embryogenesis, suggesting functional conservation of core regulators across taxonomic boundaries (Yang et al., 2023). Despite the strength of these strategies, direct translation of findings from model systems to recalcitrant trees remains a significant challenge. For example, overexpression of *Arabidopsis WUS* failed to stably induce somatic embryogenesis in *Picea abies*, underscoring species-specific differences in regulatory networks and the need for *in situ* functional validation in forest trees (Klimaszewska et al., 2010).

Following the identification of candidate DEV genes, elucidating the molecular mechanisms by which they promote somatic regeneration and transformation in recalcitrant forest species becomes essential. These mechanisms operate at multiple levels: First, DEV genes activate stem cell-related pathways to promote cellular dedifferentiation and callus formation. Members of the *WOX*, *SHOOT MERISTEMLESS* (*STM*), and *CUP-SHAPED COTYLEDON* (*CUC*) families are central regulators of stem cell identity. In *Cunninghamia lanceolata*, for instance, the peptide hormone phytosulfokine (PSK) was shown to induce embryogenic callus formation by modulating reactive oxygen species (ROS) levels and activating somatic embryogenesis (SE)-related genes (Hao et al., 2023). Similarly, plant growth regulators have been reported to promote dedifferentiation by reprogramming mature plant cells into embryogenic cells (Asghar et al., 2023). Second, DEV genes contribute to shoot meristem development by regulating bud differentiation and elongation. In *Oryza sativa*, overexpression of *BBM1* upregulates *YUCCA* genes involved in auxin biosynthesis, leading to localized auxin accumulation and shoot formation—even in the absence of exogenous hormones (Khanday et al., 2020b). Third, endogenous inhibitory pathways may pose significant barriers to regeneration, particularly in recalcitrant species. Defense-related gene activation can suppress embryogenic potential; however, such repression can be reversed by DEV gene function. In *Quercus suber*, treatment with the DNA demethylating agent 5-azacytidine reactivated SE-associated genes and alleviated epigenetic repression, thereby restoring regenerative capacity (Carneros et al., 2024). Additionally, DEV genes act within hormonal signaling networks to fine-tune phytohormone synthesis, transport, and perception, creating a microenvironment conducive to cell fate transition. For instance, auxin promotes lateral root formation via *KNOX* gene activation, while abscisic acid (ABA) often exerts antagonistic effects under certain conditions. In rice, specific *WOX* genes directly repress gibberellin (GA) biosynthesis to prevent excessive shoot elongation (Tadege, 2016). These hormonal signaling pathways are intricately interwoven, and DEV genes modulate both their expression and sensitivity, ultimately shaping favorable hormonal gradients for regeneration. This complexity also presents potential engineering targets for precise regenerative control. Taken together, mechanistic dissection of DEV gene function offers profound insights into the molecular basis of plant cell fate determination and opens new avenues for enhancing genetic transformation and regeneration efficiency in recalcitrant forest species. Looking forward, interdisciplinary strategies—integrating developmental biology, genomics, epigenetics, and synthetic regulation—will be necessary to further uncover hidden DEV regulators and resolve key biological barriers, paving the way for the rapid propagation and improvement of elite forest germplasm.

These studies collectively reveal that DEV genes such as *WUS*, *WOX*, and *KNOX* are intricately intertwined with key plant hormone signaling pathways, forming complex feedback and feedforward regulatory circuits that orchestrate plant developmental transitions (**Table 3**). However, the depth of mechanistic understanding varies among studies. While some, such as Tadege, provide direct molecular evidence including gene binding and hormone regulation, many others rely on correlative expression data without functional validation. Most existing work is also confined to model systems or highly controlled experimental conditions, underscoring the urgent need for broader cross-species analyses—particularly in recalcitrant trees such as oaks or in crop species with inherently low regenerative capacity. Moreover, the integration of emerging technologies such as single-cell transcriptomics remains limited. These approaches hold significant promise for dissecting cell-type-specific hormone–gene regulatory dynamics at high resolution. Expanding studies beyond seedling stages and under variable stress conditions may further illuminate key developmental transitions and uncover latent regulatory interactions that are critical for successful regeneration in diverse plant systems.

**Table 3:** Interaction between hormones and DEV genes.

Author/year		Plant species		Hormone		Regulatory genes		Interaction type		Key findings	
Souček et al. (2007)		*Arabidopsis*		IAA, CTK, GA, ABA		*KNOXI* (*BP*)		IAA (+), ABA (-)		IAA upregulates BP expression (associated with lateral root initiation), while ABA strongly represses BP; CK shows organ-specific regulation of *KNOXI* gene expression (Souček et al., 2007).	
Souček et al. (2007)		*Arabidopsis*		IAA, CTK, GA, ABA		*BP* (*BREVIPEDICELLUS*)		IAA (+), ABA (-)		Confirmed *BP* expression is increased by IAA and decreased by ABA, but no major effect of short-term CK or GA treatments; *BP* overexpression altered sensitivity to CK and ABA (Souček et al., 2007).	
Tadege (2016)		*Oryza sativa* (Rice)		GA		*OsWOX3A*		GA (-)		*OsWOX3A* induced by GA_3_; *OsWOX3A* directly represses GA biosynthesis via *KAO* gene, establishing negative feedback; overexpression leads to dwarfism due to reduced GA levels (Tadege, 2016).	
Su et al. (2015)		*Arabidopsis*		IAA, CTK		*WUS, WOX5*		IAA (+), CTK (+/-)		Early *WUS* and *WOX5* expression induced in embryonic callus; CK signals positively correlated with *WOX5* expression during somatic embryo formation, but negative effects via *ARR7*/*ARR15* overexpression (Su et al., 2015).	
Tvorogova et al. (2013)		General		IAA, CTK, GA		*KNOX, WUS*		IAA (+), CTK (+)		Reviewed that auxin and CK interact synergistically and antagonistically with *KNOX* and *WUS* genes, shaping meristem activity and patterning (Tvorogova et al., 2013).	
Mantegazza et al. (2009)		*Streptocarpus rexii*		GA, CTK		*SrWUS, KNOX*		GA/CTK (+/-)		Exogenous GA and CK modify anisocotyly and reposition meristematic cells; hormone crosstalk relocalizes *SrWUS* and *KNOX* expression patterns, highlighting spatial-temporal hormone-gene coordination (Mantegazza et al., 2009).	
Álvarez et al. (2020)		*Pinus pinaster*		CTK, IAA		*PpWUS*, *PpWOX*,		CTK (+/-), IAA (+/-)		A-induced *WOX* and *KNOX* genes are activated during caulogenesis; cytokinins play a triggering role (Alvarez et al., 2020).	
Zhang et al. (2023)		*Phoebe bournei*		IAA, ABA, CTK, JA		*PbWOX*		IAA (+/-), CTK (+/-), JA (+/-)		*WOX* genes show stage-specific expression during somatic embryogenesis and respond to multiple hormones (Zhang et al., 2023).	
Moore et al. (2015)		*Arabidopsis*		IAA, CTK, GA, ABA		Various		Complex		Modeled hormone gradients showing synergistic and antagonistic interactions between hormones and gene expression; identified feedback loops in hormone-gene crosstalk (Moore et al., 2015).	

Analysis of **Table 4** highlights the diverse strategies employed across plant species to promote regeneration through regulatory gene activity. Notably, regeneration-associated genes in *Quercus glauca* remain largely uncharacterized in mainstream genomic databases, underscoring a critical gap and presenting a promising direction for future research on recalcitrant *Fagaceae* species. Overall, recalcitrant forest species face a series of common challenges in somatic regeneration and genetic transformation, including: (i) difficulty in inducing and maintaining callus tissue; (ii) low regeneration efficiency coupled with high phenotypic variation among regenerated plantlets; (iii) pronounced genotype dependence, particularly in mature explants and elite cultivars; and (iv) low transformation efficiency, strongly influenced by epigenetic state and hormone metabolism. At the molecular and physiological levels, imbalances in endogenous phytohormones, epigenetic modifications (e.g., DNA methylation and histone modifications), and the regulation of key transcription factors—most notably DEV genes—have all been implicated. However, systematic functional validation of these components remains largely lacking across many forest species. Recent studies have suggested that small molecules, such as epigenetic inhibitors and antioxidants, may enhance somatic embryogenesis (SE) efficiency, offering a promising direction for intervention. Addressing these universal barriers will require the development of innovative molecular strategies tailored to the unique biological contexts of recalcitrant woody plants.

**Table 4:** Comparison of regulatory strategies and effects in different species.

Author/ year	Plant species	Target gene/pathway	Strategy adopted	Main applications and outcomes
Islam et al. (2023)	Arabidopsis	*WOX9A*, *LEC2*, *PGA37*, *WIP5*, *PEI1*, *AIL1*	Identification of key regenerative regulators via public transcriptomic data and TF network construction	Key transcription factors (TFs) and their interaction networks involved in regeneration were identified, offering potential targets for future regeneration control (Moore et al., 2015).
Tadege (2016)	*Oryza sativa* (Rice)	*OsWOX3A*	Overexpression and feedback regulation	*OsWOX3A* upregulation promotes early regeneration, while feedback inhibition of GA biosynthesis prevents excessive elongation (Tadege, 2016).
Salmon et al. (2014)	*Salix* (Willow)	*SxMAX4*	Functional complementation + QTL-based association analysis	Polymorphisms in *SxMAX4* affect adventitious bud regeneration and sprouting traits, providing molecular tools for breeding (Salmon et al., 2014).
Ding et al. (2023)	*Populus (Poplar)*	Multiple regeneration-associated genes *(REGs)* and *REGHs*	Multiple regeneration-associated genes (*REGs*) and *REGHs*	Identified 180 *REGs* and their allelic variations, revealing conserved and divergent regulatory networks between *Populus* and *Arabidopsis* (Ding et al., 2023)
Bao et al. (2009)	*Populus (Poplar)*	*588TFs*(auxin,CK,celldivision)	Genome-wide transcriptome analysis	Demonstrated that auxin/cytokinin signaling pathways and key *TFs* play critical roles during early poplar regeneration (Bao et al., 2009).
Matsuo and Banno (2008)	*Arabidopsis*	*ESR1*	*ESR1* overexpression	Promotes shoot regeneration during *Arabidopsis* tissue culture, emphasizing its transcriptional activation function (Matsuo and Banno, 2008).
Dai et al. (2021)	*Arabidopsis*	*FWA*, *WOX9*	Epigenetic modification (methylation of the *FWA* promoter)	Demonstrated that *FWA* promoter methylation regulates regeneration potential via *WOX9* (Dai et al., 2021).
Motte et al. (2014)	*Arabidopsis*	*RPK1*	QTL mapping and mutant analysis	Identified *RPK1* as a key regulator of regeneration capacity and its association with ABA signaling (Motte et al., 2014).
Tvorogova et al. (2013)	Multi-species review	*KNOX*, *WUS*	Hormone-gene interaction	Clarified the synergistic and antagonistic roles of auxin, cytokinin, and *KNOX/WUS* in regeneration control (Tvorogova et al., 2013).

## 4 Strategic pathways for DEV-gene-driven breakthroughs in somatic regeneration and genetic transformation of recalcitrant forest species

Building on more than a dozen studies across diverse forest taxa, this section synthesizes the key bottlenecks and transformative potential of DEV gene-based interventions in overcoming somatic regeneration and transformation barriers. Traditional somatic embryogenesis (SE) and transformation protocols in recalcitrant trees—such as *Pinus*, *Quercus*, and *Eucalyptus globulus*—are frequently impeded by low callus induction rates, widespread tissue browning, and poor embryo maturation and germination, rendering these systems particularly challenging for biotechnological improvement (Alvarez et al., 2013; Andrade et al., 2011; Montalbán et al., 2010). Compounding these issues are elevated endogenous levels of phenolic compounds and disrupted hormonal signaling—especially in the auxin and cytokinin pathways—which further intensify regenerative constraints (Capote et al., 2019). For instance, *Quercus* subspecies exhibit pronounced genotype dependence during SE, characterized by transcriptional repression of key embryogenic regulators such as *AINTEGUMENTA*-like and *PLETHORA*, along with accumulation of stress-related secondary metabolites (Capote et al., 2019). Additionally, robust epigenetic barriers—namely dynamic DNA methylation and histone modifications—are tightly linked to developmental recalcitrance in *Quercus suber* and related species, often manifesting as abnormal or asynchronous embryogenesis (Carneros et al., 2024; Pérez et al., 2015).

Recent findings underscore the transformative potential of DEV genes—such as *WUS*, *BBM*, and the *GRF–GIF* complex—in directly promoting embryogenic capacity and cellular proliferation, even within highly recalcitrant tissues. For example, overexpression of *HbGRF4* or the chimeric *HbGRF4–GIF1* construct markedly enhanced somatic embryo formation in *Hevea brasiliensis*, demonstrating that DEV genes can bypass genotype restrictions by activating critical transcriptional cascades (Luo et al., 2024). Similarly, in *Larix kaempferi*, overexpression of *LaCDKB1;2* improved both the quality and yield of somatic embryos through regulation of cell cycle progression, reinforcing the notion that DEV genes serve as master regulators of embryogenesis (Kong et al., 2020). Although direct functional studies of DEV genes in *Quercus velutina* (black oak) remain scarce, transcriptomic analyses of *Q. suber*—a closely related subspecies—have identified potential homologs of key embryogenesis regulators (Capote et al., 2019), paving the way for targeted interventions across other members of the *Fagaceae*.

**Figure 1:**
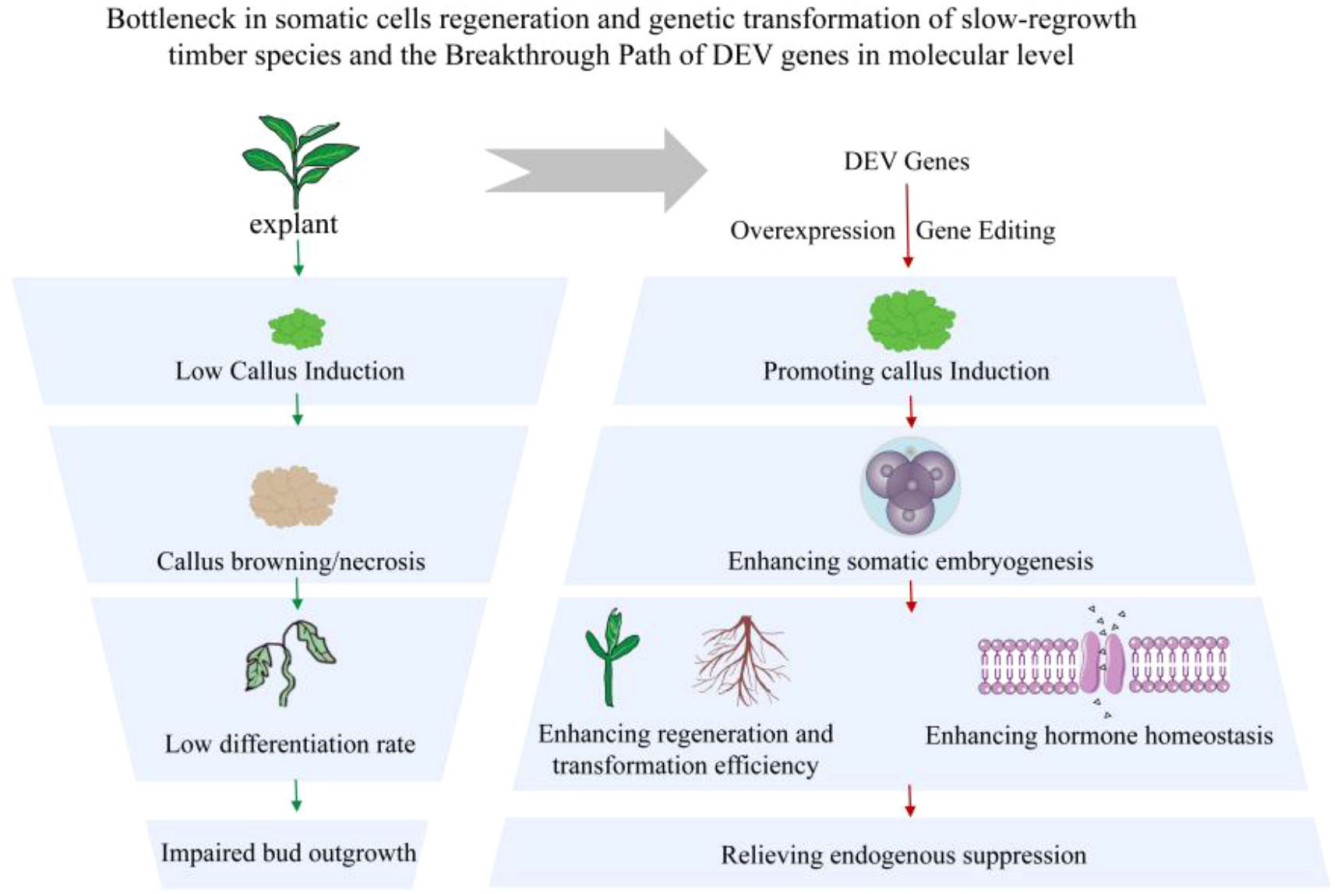
Schematic illustration of bottlenecks in somatic regeneration and genetic transformation of recalcitrant forest trees and breakthrough pathways mediated by DEV genes.

To visually summarize the typical obstacles encountered during somatic regeneration and genetic transformation in recalcitrant forest species—and to illustrate how DEV genes can overcome these barriers at the molecular level—we constructed a conceptual diagram (**Figure 1**). This figure clearly contrasts the bottlenecks in traditional regeneration pathways with the breakthrough strategies enabled by targeted DEV gene intervention. On the left side of **Figure 1**, major bottlenecks in conventional regeneration protocols are illustrated, particularly those limiting the improvement of recalcitrant tree species such as *Quercus glauca*. Beginning with the explant stage (e.g., stem segments), traditional regeneration is often constrained by a low callus induction rate, where explants fail to efficiently dedifferentiate and initiate callus formation, resulting in an early developmental blockade. Even when callus is induced, it frequently suffers from browning or necrosis, caused by cellular stress, phenolic accumulation, or oxidative damage. Subsequently, calli that do survive often exhibit low differentiation potential, struggling to develop into adventitious shoots or somatic embryos. Further along the pathway, even successfully formed shoots may exhibit poor elongation, impeding the regeneration of complete plantlets. A pervasive issue across species is genotype dependence, wherein only a narrow subset of genotypes can regenerate successfully, while the majority remain recalcitrant. These compounding constraints ultimately result in ow transformation efficiency, severely limiting the ability to generate transgenic forest plants for functional or trait-improvement purposes. In contrast, the right side of **Figure 1** presents the “Breakthrough Path” enabled by DEV gene manipulation. Through overexpression or genome editing of key DEV genes, cellular fate decisions during regeneration and transformation can be directly reprogrammed. Specific classes of DEV genes such as *WOX*, *STM* (*SHOOT MERISTEMLESS*), and *CUC* (*CUP-SHAPED COTYLEDON*) are known to promote dedifferentiation and meristem establishment; their activation significantly enhances callus induction, enabling efficient conversion of explant cells to callus tissue. Embryogenesis-related regulators such as *LEC* (*LEAFY COTYLEDON*) and *BBM* can promote somatic embryogenesis by directly inducing the formation of embryonic structures from callus, thereby bypassing the more complex organogenic routes. Additionally, genes such as *GRF–GIF* (*GROWTH-REGULATING FACTOR* – *GRF-INTERACTING FACTOR*) and *RKD4* (*RELATED TO ABI3/VP1*) have been shown to enhance overall regeneration and transformation efficiency, likely through their roles in maintaining stem cell activity, promoting cell proliferation, and coordinating cell fate transitions.

DEV genes not only reprogram cell fate but also optimize the endogenous hormonal landscape to support regeneration. By regulating key components of hormone biosynthesis and transport—such as *PIN* genes encoding auxin efflux carriers and *IPT* genes involved in cytokinin synthesis—DEV genes help establish a favorable hormonal microenvironment. Moreover, through targeted manipulation or editing of negative regulators like *VAL*, they may release intrinsic repression mechanisms that restrict regenerative competence in forest species. Collectively, DEV gene overexpression functions as a molecular switch to overcome regeneration bottlenecks: it promotes callus induction via *WUS*, *STM*, and *CUC*; sustains stem cell activity through *WOX* and *GRF–GIF*; modulates auxin–cytokinin crosstalk through *PIN* and *IPT*; and alleviates epigenetic constraints via coordinated activation of chromatin remodeling factors and transcriptional regulators (Carneros et al., 2024; Pérez et al., 2015). This integrative, DEV-centered approach represents a transformative solution for enhancing somatic regeneration and enabling stable genetic transformation in recalcitrant forest trees—an outcome long considered unattainable by conventional methods.

## 5 Identification strategies and mechanistic exploration of DEV genes in recalcitrant forest species

### 5.1 Optimizing DEV gene identification strategies to overcome regeneration recalcitrance

A comprehensive evaluation of DEV gene discovery strategies in forest trees—and their mechanistic links to overcoming somatic embryogenesis (SE) recalcitrance—highlights the central importance of transcriptomics, epigenomics, genome-wide association studies (GWAS), QTL mapping, and homology-based approaches. Comparative transcriptomic analyses, such as those conducted in *Quercus suber* (a subspecies of *Q. velutina*), have proven to be a core method. Differentially expressed transcription factors—including *AINTEGUMENTA*-like, *PLETHORA*, and *AUXIN RESPONSE FACTORs*—were identified as potential regulators by comparing gene expression profiles across key SE developmental stages, from proembryogenic masses to cotyledonary embryos (Carneros et al., 2024). These findings underscore the importance of dynamic, stage-specific expression of DEV genes, which may underlie genotype-specific recalcitrance during narrow developmental windows. Epigenomic profiling also plays a critical role in elucidating SE bottlenecks. For instance, in *Q. suber*, DNA methylation levels decrease during SE induction but increase during embryo differentiation—suggesting that dynamic methylation patterns are linked to embryogenic potential. Treatment with the demethylating agent 5-azacytidine significantly improved SE yield, while upregulation of genes such as *QsSERK1*-like further confirmed the regulatory significance of epigenetic remodeling during SE (Carneros et al., 2024). These results demonstrate the value of integrating transcriptomic and epigenomic analyses to identify key DEV gene targets in recalcitrant species.

GWAS and QTL mapping represent additional powerful strategies to associate genotype-dependent regenerative traits with genomic regions harboring candidate DEV genes. In hybrid *Populus*, allele-specific transcriptional regulation was shown to be a major contributor to differences in SE efficiency, highlighting the need for population-scale approaches to dissect the genetic basis of recalcitrance (Ding et al., 2023). Homology-based cloning and gene family analyses, as demonstrated in *Pinus* and *Picea*, offer further insights into the conservation and diversification of DEV gene families. Notably, members of the *GRAS* transcription factor family, including *SCARECROW*-like and *SHORT-ROOT* genes, were found to accumulate during the SE phase in *Pinus*, suggesting their involvement in embryogenic competence (Hernández et al., 2011). Despite significant progress, extrapolating findings from model species such as *Arabidopsis* to recalcitrant forest trees remains challenging. For example, overexpression of *Arabidopsis WUS* in *Picea glauca* failed to induce stable SE, indicating that species-specific regulatory differences may limit the functional transferability of candidate genes (Klimaszewska et al., 2010). Taken together, these strategies point toward an integrative framework in which comparative transcriptomics, epigenetic landscape profiling, GWAS/QTL mapping, and gene family homology converge to enable robust DEV gene identification and functional validation. Ultimately, this multi-dimensional approach aims to resolve the complex regulatory networks underlying SE recalcitrance and pave the way for targeted molecular interventions—such as overexpression of key transcription factors or modulation of hormonal pathways—to enhance regeneration efficiency in recalcitrant forest tree species.

### 5.2 Exploring the mechanistic roles of DEV genes in promoting somatic regeneration and genetic transformation of recalcitrant forest trees

An integrative assessment of the roles of DEV genes in promoting somatic embryogenesis (SE) and genetic transformation in recalcitrant forest species suggests that these genes operate through multiple, converging pathways. These include the induction of dedifferentiation and callus formation, stimulation of shoot differentiation and elongation, alleviation of intrinsic regeneration suppression, optimization of hormonal balance and signaling, and enhancement of transformation efficiency. Comparative transcriptomic and functional studies across diverse species have underscored the central role of DEV genes—such as *WUS*, *BBM*, and members of the *GRAS* family (e.g., *SCARECROW*)—in orchestrating cell reprogramming processes that enable otherwise non-responsive explants to bypass developmental constraints (Carneros et al., 2024; Khanday et al., 2020a). In particular, *WUS* and *WOX2* have been shown to maintain stem cell identity and establish pluripotency during embryogenic induction, especially in recalcitrant genotypes. In conifer species and *Quercus suber*, exogenous application of signaling molecules such as phytosulfokine (PSK) has been shown to trigger embryogenic callus formation by modulating reactive oxygen species (ROS) levels and subsequently activating SE-associated gene expression (Hao et al., 2023). These findings support the hypothesis that DEV genes interact with hormonal and redox signaling pathways to remodel cell fate and promote regenerative competence in difficult-to-transform forest trees.

Evidence from diverse plant systems suggests that regenerative reprogramming is achieved through a convergence of transcriptional regulation, hormonal crosstalk, and epigenetic remodeling. At the transcriptional level, developmental regulators reactivate stem cell programs and initiate cascades that drive cellular dedifferentiation, establishment of embryogenic competence, and subsequent organogenic transitions. These transcriptional events are closely integrated with phytohormone dynamics, where auxin–cytokinin interactions promote organ initiation, while antagonistic signals such as ABA and GA impose developmental constraints that must be relieved to sustain regeneration. Epigenetic modifications, particularly changes in DNA methylation and chromatin accessibility, further determine the responsiveness of cells by shaping the accessibility of key regulatory loci. Rather than operating independently, these mechanisms form an interdependent network that resets cellular identity and stabilizes regenerative trajectories. This mechanistic framework, consistently observed across model and crop species, provides a conceptual foundation for adapting developmental gene-based strategies to the improvement of recalcitrant forest trees.

## 6 Functional validation of DEV genes and strategic advances in regeneration systems for recalcitrant forest trees

### 6.1 Challenges and approaches in the functional validation of DEV genes

Functional validation of *DEV* genes in recalcitrant forest species faces numerous inherent challenges, primarily due to their notoriously low regeneration and transformation efficiencies. To address this, multiple methodological strategies have been developed. Transient expression systems, including agroinfiltration, particle bombardment, and protoplast-based assays, provide rapid means to assess candidate DEV gene activity without requiring stable transformation. The use of reporter genes such as GFP and GUS allows spatiotemporal visualization of DEV gene expression patterns during callus induction and somatic embryogenesis, yielding insights into their roles in early developmental reprogramming (Li et al., 2025). Despite technical difficulties, stable transformation remains the gold standard for functional characterization. Progress in *Pinus pinaster* and *Quercus suber* has demonstrated that Agrobacterium-mediated transformation combined with somatic embryogenesis systems can serve as a reliable platform for gene function analysis (Hassani et al., 2022). CRISPR/Cas9-based genome editing provides additional precision for generating knockouts, allelic modifications, or controlled overexpression lines, and has already been applied in larch and other conifers (Jain, 2006; Ma et al., 2024). These approaches allow researchers to dissect the specific contribution of DEV genes to regeneration, particularly when integrated with transcriptomics, epigenomics, and protein–protein interaction assays (Zhao et al., 2022) (Capote et al., 2019). This suggests that DEV genes can serve as preparatory or co-transformation tools to enhance post-transformation regenerative capacity, thereby overcoming one of the key bottlenecks limiting genetic improvement in forest species.

### 6.2 Strategies to enhance regeneration efficiency in recalcitrant forest trees via DEV genes

This review outlines emerging strategies for improving regeneration efficiency in stress-resilient, recalcitrant forest species by leveraging DEV genes. Increasingly, approaches involving the fine-tuning of DEV gene expression, multigene coordination, integration with epigenetic regulation, and their incorporation into transformation systems are recognized as promising pathways for overcoming long-standing bottlenecks in somatic embryogenesis (SE). Studies across diverse forest and model species have demonstrated that the spatiotemporal control of DEV genes such as *WUS* and *LEAFY COTYLEDON1* (*LEC1*) plays a critical role in reprogramming embryogenic competence. For example, overexpression of *AtWUS* in *Gossypium* resulted in a threefold increase in embryogenic tissue induction, underscoring the importance of gene dosage and tissue-specific expression (Bouchabke-Coussa et al., 2013). Similarly, application of *PpWOX2* in *Pinus pinaster* revealed that precise timing and developmental context are essential for achieving optimal embryo quality and yield (Hassani et al., 2022).

Recent findings also highlight the advantages of coordinated multigene regulation. In hybrid *Liquidambar*, the interaction between *WRKY29* and *GRF2* defines a regulatory module that integrates stress signaling and developmental control, illustrating the potential of transcription factor combinations in orchestrating complex regenerative programs (Li et al., 2025). In *Picea abies*, overexpression of *PaHAP3A* during maturation induced ectopic embryogenesis, further suggesting that stage-specific activation of DEV genes may expand embryogenic capacity (Uddenberg et al., 2016). The integration of DEV gene regulation with epigenetic modulation has also proven critical. In *Quercus suber*, treatment with the demethylating agent 5-azacytidine significantly enhanced SE output, indicating that activation of DEV genes can be potentiated by relieving epigenetic constraints (Carneros et al., 2024). This approach aligns with studies in conifers, where small molecules such as histone deacetylase inhibitors have been proposed as adjunct tools to optimize SE outcomes (Guo et al., 2024). Finally, embedding DEV genes into existing transformation protocols has emerged as a key strategy for forest tree improvement. While overexpression of *WUS* alone may not fully regenerate recalcitrant *Gossypium* plants, it successfully induced embryogenic structures that serve as a platform for transformation and regeneration (Bouchabke-Coussa et al., 2013). This illustrates how DEV genes can function as “regeneration boosters” within transformation workflows, potentially enhancing both the efficiency and consistency of genetic transformation in otherwise intractable species.

## 7 DEV genes as a novel trajectory for accelerating forest tree molecular breeding

Despite the substantial promise DEV genes hold for enhancing somatic regeneration and genetic improvement in forest trees, their widespread application in forestry remains impeded by a series of formidable challenges that collectively hinder progress in molecular tree breeding. Chief among these is the intrinsic recalcitrance to regeneration that characterizes most economically and ecologically important woody species. As noted by Lelu-Walter (Lelu-Walter et al., 2013)and Izuno (Izuno et al., 2020), mature forest trees—often the most desirable targets for breeding—exhibit severe regeneration barriers under *in vitro* conditions. These include pronounced genotype dependency, wherein only a limited number of genotypes respond favorably to regeneration protocols; strong physiological age effects, such that regenerative competence is generally restricted to juvenile tissues like immature zygotic embryos or cotyledons, while mature elite genotypes exhibit extremely low or even absent somatic embryogenic potential. Moreover, these species display heightened sensitivity to plant growth regulators, rendering it difficult to define universally effective hormonal conditions. Long-term tissue culture further carries the risk of somaclonal variation, potentially compromising the genetic fidelity of regenerated lines. A representative case is *Quercus acutissima*, a valuable endemic hardwood species in China. It exemplifies typical recalcitrant features such as poor shoot elongation and early developmental arrest during culture, severely limiting the efficacy of its biotechnological breeding efforts. Beyond biological constraints, the large and highly heterozygous genomes of forest trees pose additional technical hurdles. Capote reported the abundance of repetitive sequences in tree genomes, which hampers accurate genome assembly, gene annotation, and downstream functional genomic studies (Capote et al., 2019). Compared to model herbaceous plants, functional genomics in trees remains underdeveloped due to the lack of mutant libraries and comprehensive gene function annotations, ultimately restricting the identification and validation of DEV genes in tree species. Furthermore, the extended life cycles of forest trees necessitate long-term evaluation of transgenic lines to assess traits such as growth performance, wood properties, stress tolerance, and ecological adaptability—an endeavor that is both time-consuming and costly. Perhaps more critically, Merkle (Merkle and Dean, 2000)and Lelu-Walter (Lelu-Walter et al., 2013) have underscored the persistent ethical, social, and regulatory barriers associated with the deployment of genetically modified trees. These non-technical constraints demand equal attention and proactive engagement to ensure the responsible and publicly acceptable implementation of DEV-based breeding technologies.

Despite the formidable challenges, the rapid advances in modern biotechnology and computational science have ushered in an unprecedented era of opportunity for overcoming the long-standing recalcitrance of forest trees to somatic regeneration and genetic transformation. These opportunities are driven by breakthroughs across several key technological domains. Foremost, the explosive growth of high-throughput omics technologies has provided powerful tools for dissecting the molecular underpinnings of tree regeneration. As highlighted by Pais, emerging platforms such as single-cell RNA sequencing and spatial transcriptomics offer unprecedented resolution in capturing cellular heterogeneity, lineage trajectories, and the spatiotemporal dynamics of DEV gene expression during regeneration (Pais, 2019). These technologies enable precise identification of critical DEV genes acting at specific developmental junctures—such as dedifferentiation, proliferation, or redifferentiation—thus facilitating more targeted and stage-specific molecular interventions. Second, the maturation of genome editing technologies, particularly CRISPR/Cas9, has revolutionized the precision and efficiency of forest tree improvement. As demonstrated by Li, gene editing now permits the targeted knockout, overexpression, or fine-tuning of endogenous DEV genes (Li et al., 2025). By silencing negative regulators of regeneration or activating key positive regulators in a temporally controlled manner, this approach offers a promising strategy to circumvent genotype-dependent regeneration barriers that have long hampered transformation in woody perennials. Moreover, the accelerating integration of artificial intelligence (AI) and machine learning into plant biology is beginning to reshape our ability to mine and interpret the vast multi-omics datasets generated in tree systems. Studies by Zhao (Zhao et al., 2022) have already employed large-scale data analytics to uncover gene networks associated with complex traits. In the near future, AI-driven algorithms are expected to predict optimal DEV gene combinations, optimize culture media formulations, and decode the regulatory logic underlying regeneration bottlenecks—thereby streamlining experimental design and expediting phenotypic evaluation, ultimately shortening the breeding cycle. Finally, the conceptual framework of synthetic biology is emerging as a transformative paradigm for forest tree biotechnology. By integrating multiple DEV genes, regulatory elements, and reporters into modular, controllable gene circuits, synthetic systems aim to achieve finely tuned control over regeneration processes. This engineered, systems-level approach transcends the limitations of single-gene strategies and opens the door to high-efficiency, context-specific regeneration, including the development of “smart” circuits that respond only to designated stimuli—potentially addressing biosafety concerns associated with genetically modified trees.

Looking ahead, in-depth studies and applications of DEV genes are poised to revolutionize forest biotechnology, ultimately contributing to global sustainable forestry and ecological civilization. The most immediate and impactful application lies in the establishment of robust and broadly applicable somatic regeneration systems for recalcitrant tree species. Through precise modulation of DEV gene activity—including the overexpression of key positive regulators, the CRISPR-mediated knockout or silencing of negative regulators, and the synergistic action of multiple transcription factors—it becomes feasible to overcome the regeneration bottlenecks characteristic of species such as *Quercus glauca*, achieving efficient and genotype-independent *in vitro* regeneration. This would break the long-standing constraints of genotype dependency in conventional breeding, significantly expanding the genetic resources available for tree improvement. Building upon these high-efficiency regeneration platforms, the development of mature and stable transformation systems in forest species becomes a tangible goal. DEV genes, acting as “regeneration boosters,” provide the cellular and molecular foundation necessary for the successful genetic introduction of desirable traits, including disease resistance, abiotic stress tolerance (e.g., drought, cold, salinity), rapid growth, and superior wood quality. Such advances will accelerate the genetic improvement of ecologically and economically important species like *Q. glauca*, enhancing their resilience under climate change and meeting the growing societal demand for high-value forest products. Beyond genetic enhancement, the integration of somatic embryogenesis with bioreactor technologies offers the prospect of large-scale, automated clonal propagation of elite genotypes. This will enable mass production of uniform, high-quality planting stock for afforestation, ecological restoration, and industrial plantations, dramatically improving forestry productivity and economic returns. As demonstrated by Egertsdotter, automation of somatic embryogenesis holds significant promise for scalable deployment (Egertsdotter et al., 2019). Importantly, DEV-assisted regeneration systems also provide a powerful tool for the long-term conservation of elite or endangered forest germplasm. When coupled with cryopreservation technologies, these systems enable secure and viable preservation of genetic diversity, safeguarding valuable allelic variation for future breeding programs. Most critically, accelerated tree breeding enabled by DEV gene research will support the selection and deployment of climate-resilient, high-carbon-sequestration, and ecologically beneficial tree species. This directly aligns with global targets for carbon neutrality and climate change mitigation, offering a foundational technological contribution to sustainable forest management. Finally, overcoming the complexity of forest biotechnology will require deep interdisciplinary integration and global collaboration. As emphasized by Pinto, synergistic advances across bioinformatics, molecular and cell biology, genetics, forestry, and environmental sciences—together with international partnerships—are essential to confront the formidable challenges ahead (Pinto et al., 2013). Only through such collective efforts can we realize the full potential of DEV genes in reshaping forest biotechnology and usher in a new era of climate-smart, sustainable forestry.

## 8 Conclusion

Research on DEV genes has fundamentally reshaped our understanding of plant cellular totipotency and fate reprogramming, demonstrating remarkable potential for enhancing regeneration and transformation efficiency in both model and crop species. For recalcitrant forest trees, DEV genes represent a pivotal breakthrough for overcoming long-standing bottlenecks in somatic regeneration and genetic transformation. Although these species present considerable challenges—including genomic complexity, low transformation efficiency, and functional validation difficulties—emerging technologies such as high-throughput multi-omics, genome editing, and artificial intelligence are opening unprecedented opportunities. Looking ahead, synthetic biology approaches may enable the construction of modular regulatory circuits for precise spatiotemporal control, while AI-assisted data mining can accelerate DEV gene discovery and optimize regeneration conditions. Coordinated multigene regulation, rather than single-gene manipulation, will be critical for tackling complex developmental barriers. Equally important are biosafety considerations, ensuring that DEV-based innovations in forestry proceed responsibly and sustainably. In this context, the systematic identification, functional dissection, and regulation of DEV genes hold promise for establishing robust regeneration and transformation systems in recalcitrant species such as *Quercus glauca*. Such advances will accelerate genetic improvement, enable large-scale clonal propagation, and ensure effective germplasm conservation. Ultimately, these efforts will provide critical technological support for the sustainable development of forestry and the advancement of global ecological civilization.
